# The Cry for Freedom

**Published:** 1918-08-31

**Authors:** 


### A NURSES' CHARTER OF LIBERTY.

### The Cry for Freedom.

I welcome the opportunity of replying to the views expressed by "Matron" in your issue of the 24th instant. I have heard those views before. I heard them when I was a child in my own home in connection with domestic servants. It is not long since an old lady friend of mine boasted to me in a very modest way—which may be a paradox—of her motherly care of her maid. The girl had come from a country village, and her mistress very kindly escorted her to town on her "off" afternoon, waited for her while she looked at the shop windows, and brought her safely home again. With the addition of a chain it was precisely how she mothered her Pekinese. I ask the impartial observer to look around and note the result of this kind of benevolent despotism. The domestic servant famine is sore in the land, and whether it is good for them or not girls will have none of it. From the depths of the human soul comes the cry for freedom. It is a passion that burns more fiercely than all others, and its suppression in the case of nurses accounts for their shortage, and for hospitals, especially those of second grade, having to lower the standard for recruits. It is, in fact, the domestic servant problem repeating itself.

### Nurses' Views Clearly Expressed.

"Matron" thinks I imagine all this. Let me quote from my mail-bag.

"Eight hours a day is desirable, and I think a better class of woman would be induced to train if nurses could 'live out.' The restrictions of the Nurses' Home are too irksome to the majority." (Matron in the Midlands.)

"When one is working at full pressure and the difficulty is to get all the duties done and when every letter written means so much time taken from sleep, it is difficult to get even the ten minutes the Editor speaks of to state one's views." (District Nurse.)

"All institutions must wake to the fact that they are dealing with women, not schoolgirls or machines, and regulations must be less one-sided. More recreation is a great need for workers whose lot is frequently of necessity depressing and nerve racking." (Provincial Sister.)

"I consider eight-hour days quite sufficient for hospital nurses: relief should be arranged for night nurses, so that they should be able to take their meals right away from the wards. Nurses often do without meals in the night as they cannot find time for same." (Member of the College of Nursing.)

"Not more than forty-four hours per week: one day off per week: five weeks' holidays per year. Trained hospital nurses to be allowed to live out if they so desire. Salary for fully trained nurses at least £150 or its equivalent, with £20 for each extra certificate, such as C.M.B., R.S.I., Massage." (Manchester Nurse.)

"Nurses' hours are much too long, both day and night duty. An eight-hour day would in my opinion solve this problem. Nurses should have *at least* four weeks' holiday each year, and if possible one additional week in the spring-time." (Member of College of Nursing.)

"Eight hours per day. For well-being of all nurses this is very desirable, but the hours of work should be consecutive, say from 6 A.M. to 2 P.M.; 2 P.M. to 10 P.M.; 10 P.M. to 6 P.M. Four hours on duty, fours hours off, and then four hours on again takes away a nurse's interest in her cases. If nurses worked only eight hours per day, stated hours for study could be set apart in their off-duty time." (Hampstead Sister.)

"I support with all my heart an eight-hour day for all nurses: better salaries for sisters: increased time daily off duty for senior sisters. I trust all trained nurses will interest themselves in this matter and believe that the College of Nursing will also move in supporting this. Nurses will never get reasonable privileges and reasonable hours of duty until they unite and work for them." (Argyllshire Nurse.)

"I have had many years of nursing, and I am only just beginning to realise what idiots we nurses are. We work for a mere pittance and little thanks. No profession is so abominably paid, and yet the majority are afraid to speak." (Provincial Nurse.)

"Many nurses consider their hours of duty excessive but fear to compromise themselves by giving their names." (London Hospital Nurse.)

### Let the Public Know.

Shall I continue to quote, or will this batch taken at random suffice to convince "Matron" that it is she who is out of touch with the mind of the workers? "Matron's" letter will also convince nurses of the truth of what I have been endeavouring to impress upon them, that their silence is apt to cause them to be misunderstood.

As regards remuneration this is a subject which I have not space this week to discuss. But as to how the money is to be found to enable hospitals to provide the extra necessary accommodation and funds I should like in reply to ask "Matron" whether or not she realises that the nursing of the nation's poor is public service of priceless value to the Empire? Does she think if it were put to the British people that hospital nurses discharging this duty are overwrought and underpaid that they would hesitate to supply the money? I do not. The public are unaware of the prevailing discontent, and how can they be otherwise? "Matron" is unaware of it, and the nurses' silence is interpreted as a sign and a token that they "are quite happy in the lot they have chosen."

### Is the Profession Being Deserted?

It is to provide a remedy that ten thousand women have joined the College of Nursing, and by doing this it is obvious that hospitals as well as nurses will derive substantial benefit. It is erroneous to suppose that nurses will not know what to do with their leisure. What do the myriads of women clerks in the Post Office who work a seven-hour day, and in banks and insurance offices, do with their leisure? Do they get into mischief? My own view is that the suggestion is too absurd to discuss. But I think it is well worthy of discussion whether or not the nursing profession is being deserted for other callings where the hours of duty are less oppressive, and whether women of culture and refinement from whose ranks recruits have come in the past will not elect to turn their attention to more alluring employment.

